# PET/CT Imaging in Oncology: Exceptions That Prove the Rule

**DOI:** 10.1155/2013/865032

**Published:** 2013-02-27

**Authors:** M. Casali, A. Froio, C. Carbonelli, A. Versari

**Affiliations:** ^1^Nuclear Medicine Unit, Department of Advanced Technology, Azienda Ospedaliera Santa Maria Nuova, IRCCS, Viale Risorgimento 80, 42123 Reggio Emilia, Italy; ^2^Pulmonology Unit, Department of Cardiology, Thoracic and Vascular Surgery and Critical Care Medicine, Azienda Ospedaliera Santa Maria Nuova, IRCCS, Viale Risorgimento 80, 42123 Reggio Emilia, Italy

## Abstract

^18^F-FDG PET/CT is a diagnostic three-dimensional non-invasive device, routinely employed in neurology, cardiology, and oncology, and which contributes to patient care giving functional informations about glucose metabolism. In particular, staging, restaging, follow-up and response to treatment of tumors are the most common applications in oncologic field. Many neoplasms show increased glucose metabolism and consequent ^18^F-FDG uptake. Nevertheless, some relative differentiated cancers, such as clear cell carcinoma of the kidney and bronchioloalveolar adenocarcinoma, show tipically faintly/no uptake resulting in a consequent negative PET/CT scan. This case report represents an extreme case in which three relative well-differentiated cancer forms, all characterized by low glucose metabolism, affect the same patient at the same time while ^18^F-FDG PET/CT scan is negative.

## 1. Introduction

^18^F-FDG is an established agent for detecting and staging tumors [1, 2]. However the most common limits of this technique are represented by well-differentiated cancer forms and in general tumors with low proliferative index [2]. The uniqueness of this case report consists in the concurrence of three metachronous cancers all characterized by low glucose metabolism and a consequent negative PET/CT study. 

## 2. Case Report

**Figure 1:**
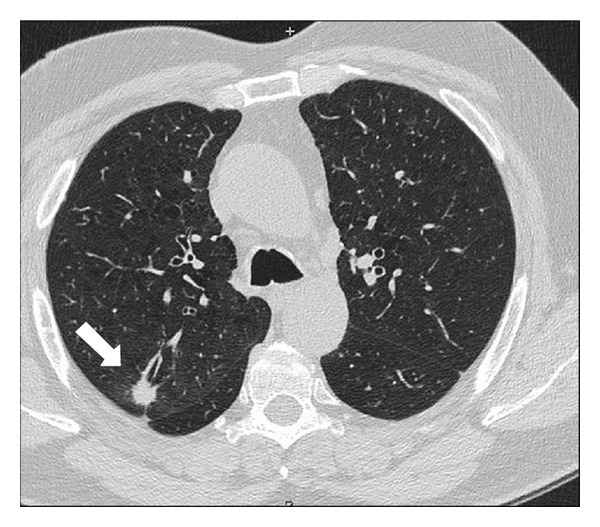
Accidental nodule of the right pulmonary upper lobe (arrow).

**Figure 2:**
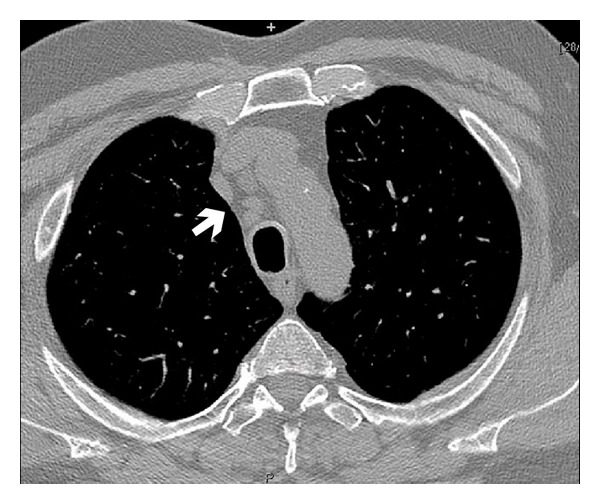
Mediastinal lymphoadenopathies (arrow).

**Figure 3:**
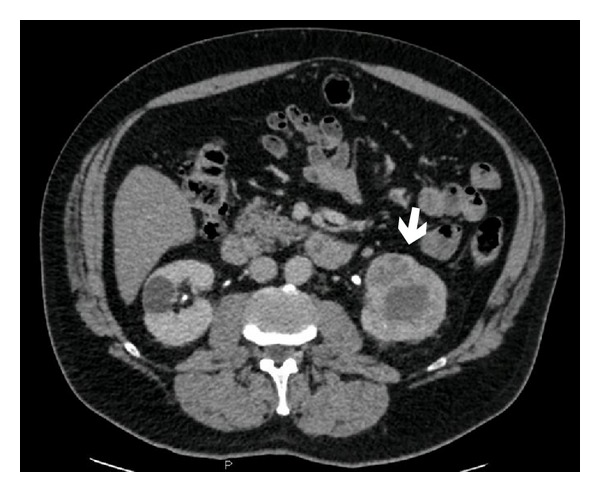
Accidental radiological finding of left renal mass (arrow).

**Figure 4:**
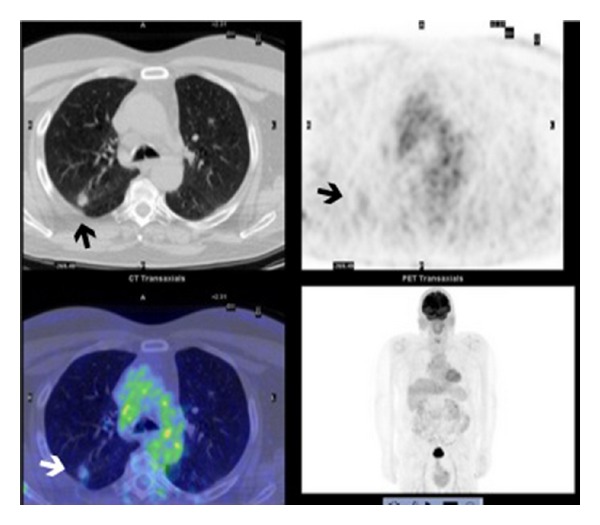
Faintly ^18^F-FDG uptake of the right pulmonary upper lobe—SUVmax 1.3 (arrow).

**Figure 5:**
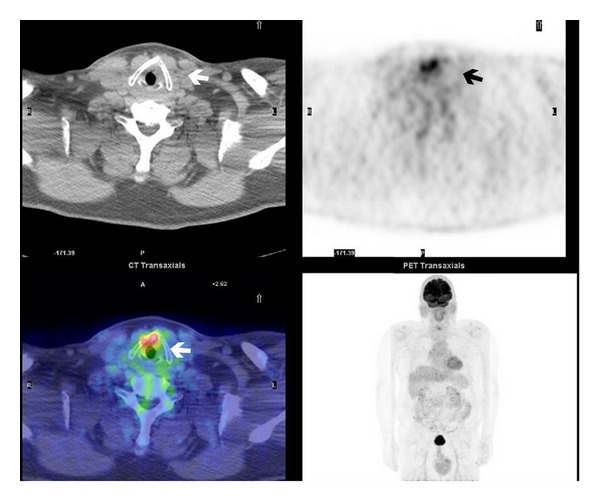
Asymmetrical ^18^F-FDG uptake appreciable in larynx at PET/CT scan (arrow).

**Figure 6:**
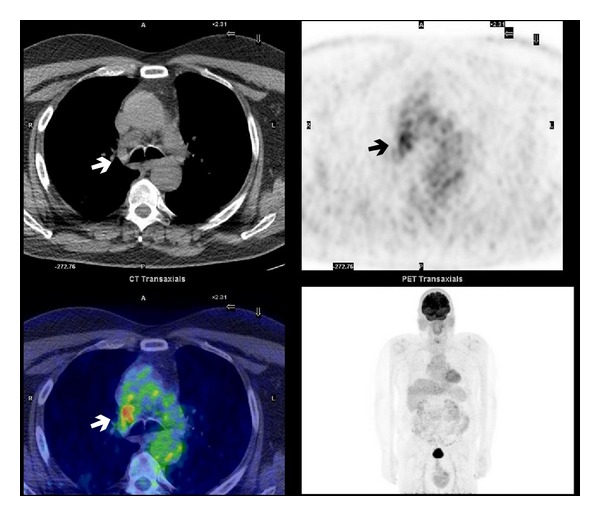
Right paratracheal suspicious lymphadenopathy at PET/CT scan—SUVmax 4.6 (arrow).

Patient: male, 65 yrs old. In December 2011th underwent an ENT visit because of recent onset of dysphonia in smoking status (20 cigarettes/die) with objective diagnosis of clear nasal passages, normal oropharynx, normal larynx motility, and leukoplakia infiltrating the anterior third of the left vocal cord. The day after the ENT visit he also underwent a chest X-ray with accidental diagnosis of right pulmonary upper lobe nodule worthy of thorough investigation with a computed tomography. CT scan of the chest confirmed nodule presence, indicating the neoplastic nature, and also lower paratracheal (right), mediastinal para-aortic, and right pulmonary hilar lymphadenopathies (Figures 1 and 2). An increase in size of left adrenal gland was also described. In January 2012 a biopsy of vocal cords was done with diagnosis of mildly differentiated infiltrating squamous cell carcinoma only for the left cord. Then an abdominal CT scan was done with accidental finding of a neoplastic mass of 6 cm of maximum diameter in the left kidney (Figure 3). At the end of January 2012, five days after left vocal cord biopsy, the patient underwent a ^18^F-FDG PET/CT scan (blood glucose level at the administration of the tracer was 87 mg/dL) with diagnosis of faint tracer uptake of the already known right pulmonary nodule (SUVmax 1.3) and also diagnosis of only one right paratracheal suspicious lymphadenopathy (SUVmax 4.6) worthy of followup (Figures 4, 5, and 6). At the beginning of February 2012 the patient was subjected to CO_2_-laser endoscopic laryngectomy. Histology confirmed the presence of mildly differentiated infiltrating squamous cell carcinoma. In February he was subjected to an atypical resection of the right upper lung nodule and to lymphadenectomy of the node identified at the PET/CT study. Bioptic examination revealed a well-differentiated lung adenocarcinoma characterized by 90% of lipid component and a remaining part of infiltrating acinous pattern, unexpectedly a mediastinal node involvement by clear cell carcinoma suspicious of renal origin. Finally, in April 2012, the patient underwent left nephrectomy with associated homolateral adrenal removal. Histology confirmed the presence of clear cell renal carcinoma and adenomatous hyperplasia of the left adrenal gland.

## 3. Discussion 

In recent years ^18^F-FDG PET/CT has worldwide demonstrated itself as one of the most important oncological diagnostic devices [3–5]. However the most common limits of this technique are represented by well-differentiated cancer forms and in general tumors with low proliferative index [6, 7]. Bronchioloalveolar lung adenocarcinoma or clear cell tumor of the kidney is the most common example of low neoplastic uptake of ^18^F-FDG [8]. The uniqueness of this case report consists in the concurrence of three metachronous cancers (mildly differentiated infiltrating squamous cell carcinoma, well-differentiated lung cancer, and clear cell renal carcinoma) all characterized by low glucose metabolism and a consequent negative PET/CT study. A retrospective evaluation of the ^18^F-FDG PET/CT scan was able to characterize only a very low uptake of the tracer by lung nodule, while no pathological uptake was seen in the left kidney. Only mildly asymmetrical uptake was appreciable in larynx (partial hypocaptation of the left vocal cord), but a few days before the patient had been subjected to vocal cord biopsy. This is an extreme case, the exceptions that prove the rule, that reminds us that ^18^F-FDG is surely an optimal tracer but not a “universal” tracer valid for all kinds of cancers and that all scenarios however unlikely are possible, keeping in mind that a true cancer diagnosis is only bioptic.
